# Parturition among the Esquimaux

**Published:** 1852-07

**Authors:** 


### OBSTETRICS.

Partutrition among the Esquimaux.—At a recent meeting of the Obstetrical Society, Dr. James Struthers exhibited the pelvis of a female Esquimaux, which was chiefly remarkable for the unusually large dimensions of the brim, cavity, and outlet, allowing the largest fœtal head to pass with great ease in almost any direction. The following are the diameters of the brim:—Transverse, 6 inches; conjugate, 4⅝ inches; oblique, 5¾ inches. Of the cavity:—Transverse, 5¼ inches; conjugate, 5⅝ inches: oblique, 5½ inches. The transverse diameter of the outlet was 5½ inches; and the public arch was unusually wide.

Dr. Struthers received the pelvis from Mr. G. C. Pirie, who found it last summer under a cairn of stones, (the common mode of burying among the Esquimaux,) near Cape Hooper, in Davies' Straits. It is an interesting fact, (of which Mr. Pirie satisfied himself, thereby confirming the statements of various writers on Greenland,) that the Esquimaux women have much safer and more rapid labors than the women of this country. There the whole process of parturition is left in the most absolute manner to nature. As soon as the woman begins to complain, she retires to a low skin hut, built for the purpose, into which no one is allowed on any account to accompany her. Before the lapse of an hour, she generally makes her appearance with the baby on her back. Not unfrequently labor comes on unexpectedly. Such a case came under Mr. Pirie's own observation. The woman left the ship, retired behind a block of ice, and came back within half an hour with the child in her hood. It is stated that the cord is not divided until the placenta has come away, and that the division is effected by the teeth of the mother. Miscarriages, twins, and monsters are of rare occurrence, and it is a very uncommon thing for a woman to die in childbed.—*Medical Times and Gazette, May 8th,* 1852.

